# *N*′-[(*E*)-2-Fluoro­benzyl­idene]benzo­hydrazide

**DOI:** 10.1107/S1600536813031747

**Published:** 2013-11-27

**Authors:** P. B. Sreeja, M. Sithambaresan, N. Aiswarya, M. R. Prathapachandra Kurup

**Affiliations:** a Department of Chemistry, Christ University, Hosur Road, Bangalore 560 029, Karnataka, India; b Department of Chemistry, Faculty of Science, Eastern University, Sri Lanka, Chenkalady, Sri Lanka; c Department of Applied Chemistry, Cochin University of Science and Technology, Kochi 682 022, India

## Abstract

The asymmetric unit of the title compound, C_14_H_11_FN_2_O, contains two independent mol­ecules, both of which adopt the *E* conformation with respect to the azomethine C=N bond. The mol­ecules are non-planar, with dihedral angles of 26.92 (12) and 11.36 (11)° between the benzene and phenyl rings. In the crystal, mol­ecules are linked through N—H⋯O=C and N—H⋯N hydrogen bonds into chains along [101]. C—H⋯O contacts link these chains into layers parallel to (001). The three-dimensional crystal packing is stabilized by π–π inter­actions, the shortest separation between the centroids of benzene rings being 3.884 (1) Å.

## Related literature

For catalytic properties of hydrazones, see: Heravi *et al.* (2007 ▶). For their use as inhibitors of enzymes, see: Tamasi *et al.* (2005 ▶) and for their biological activity, see: Sreeja *et al.* (2004 ▶). For the synthesis of related compounds, see: Mangalam & Kurup (2011 ▶). For a related structure, see: Nair *et al.* (2012 ▶).
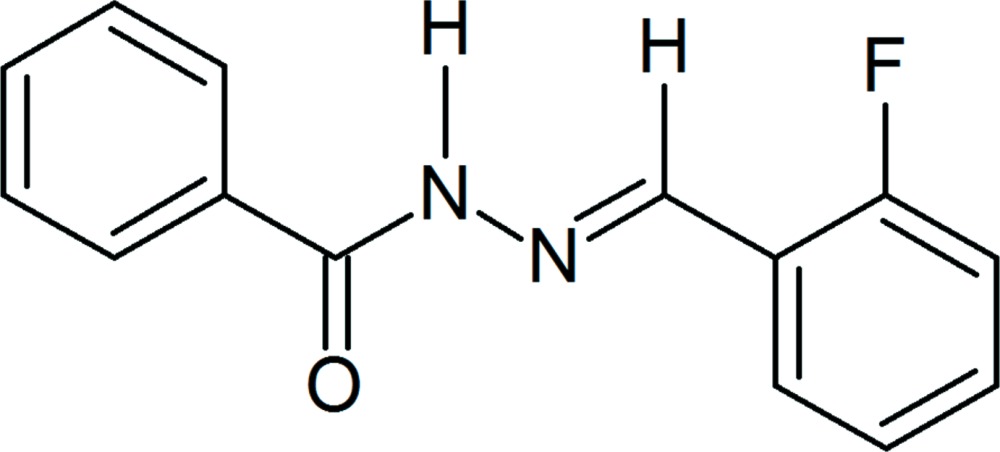


## Experimental

### 

#### Crystal data

C_14_H_11_FN_2_O
*M* *_r_* = 242.25
Monoclinic, 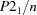

*a* = 9.7010 (6) Å
*b* = 17.4114 (13) Å
*c* = 15.002 (1) Å
β = 104.126 (4)°
*V* = 2457.3 (3) Å^3^
*Z* = 8
Mo *K*α radiation
μ = 0.10 mm^−1^
*T* = 296 K
0.35 × 0.30 × 0.25 mm

#### Data collection

Bruker Kappa APEXII CCD area-detector diffractometer
Absorption correction: multi-scan (*SADABS*; Bruker, 2007 ▶) *T*_min_ = 0.968, *T*_max_ = 0.977
18766 measured reflections
6111 independent reflections
3320 reflections with *I* > 2σ(*I*)
*R*_int_ = 0.026

#### Refinement

*R*[*F* ^2^ > 2σ(*F* ^2^)] = 0.050
*wR*(*F* ^2^) = 0.165
*S* = 1.00
5972 reflections
333 parameters
2 restraints
H atoms treated by a mixture of independent and constrained refinement
Δρ_max_ = 0.21 e Å^−3^
Δρ_min_ = −0.17 e Å^−3^

### 

Data collection: *APEX2* (Bruker, 2007 ▶); cell refinement: *SAINT* (Bruker, 2007 ▶); data reduction: *SAINT*; program(s) used to solve structure: *SHELXS97* (Sheldrick, 2008 ▶); program(s) used to refine structure: *SHELXL97* (Sheldrick, 2008 ▶); molecular graphics: *ORTEP-3 for Windows* (Farrugia, 2012 ▶) and *DIAMOND* (Brandenburg, 2010 ▶); software used to prepare material for publication: *SHELXL97* and *publCIF* (Westrip, 2010 ▶).

## Supplementary Material

Crystal structure: contains datablock(s) Global, I. DOI: 10.1107/S1600536813031747/yk2100sup1.cif

Structure factors: contains datablock(s) I. DOI: 10.1107/S1600536813031747/yk2100Isup2.hkl

Supplementary material file. DOI: 10.1107/S1600536813031747/yk2100Isup3.cml

Additional supplementary materials:  crystallographic information; 3D view; checkCIF report

## Figures and Tables

**Table 1:** Hydrogen-bond geometry (Å, °)

*D*—H⋯*A*	*D*—H	H⋯*A*	*D*⋯*A*	*D*—H⋯*A*
N2—H2′⋯O2^i^	0.89 (1)	2.09 (2)	2.875 (2)	147 (2)
N2—H2′⋯N3^i^	0.89 (1)	2.60 (2)	3.339 (2)	142 (2)
N4—H4′⋯O1	0.87 (1)	2.03 (1)	2.882 (2)	171 (2)
C7—H7⋯O2^i^	0.93	2.53	3.213 (2)	131
C14—H14⋯O2^i^	0.93	2.49	3.402 (3)	166
C16—H16⋯O2^ii^	0.93	2.55	3.450 (3)	164
C21—H21⋯O1	0.93	2.44	3.250 (2)	145
C28—H28⋯O1	0.93	2.40	3.312 (2)	166

Symmetry codes: (i) 
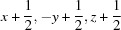
; (ii) 
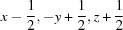
.

**Fig. 1.**
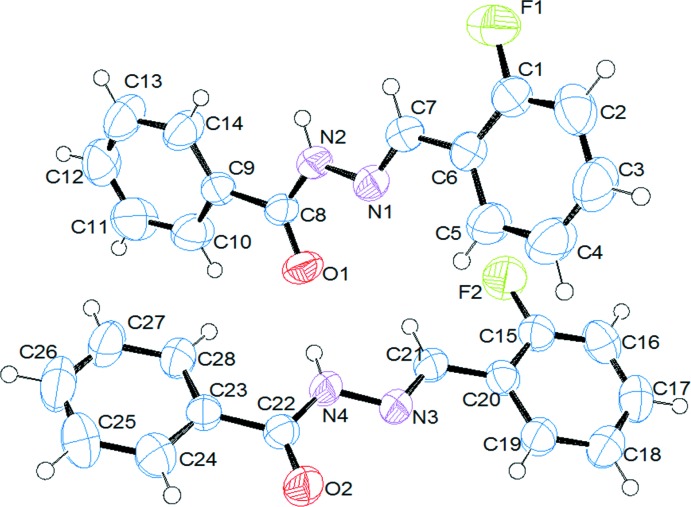
ORTEP diagram of N'-[(E)-(2-fluorophenyl)methylidene]benzohydrazide with 50% probability ellipsoids.

**Fig. 2.**
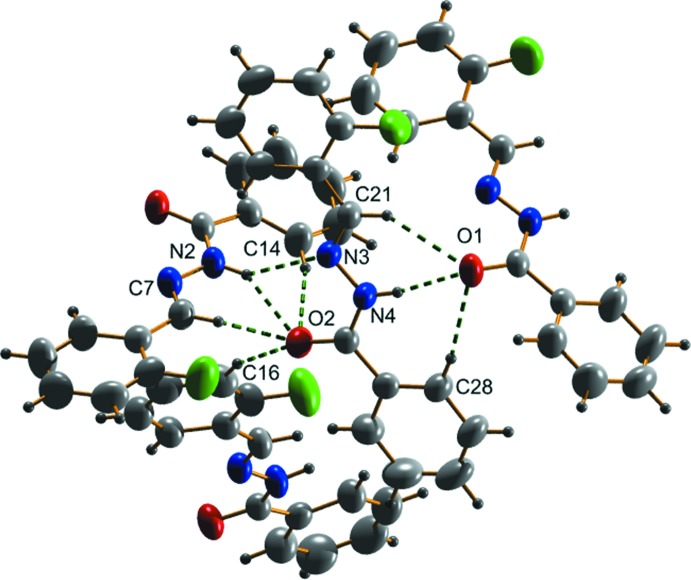
Hydrogen-bonding interactions of the title compound.

**Fig. 3.**
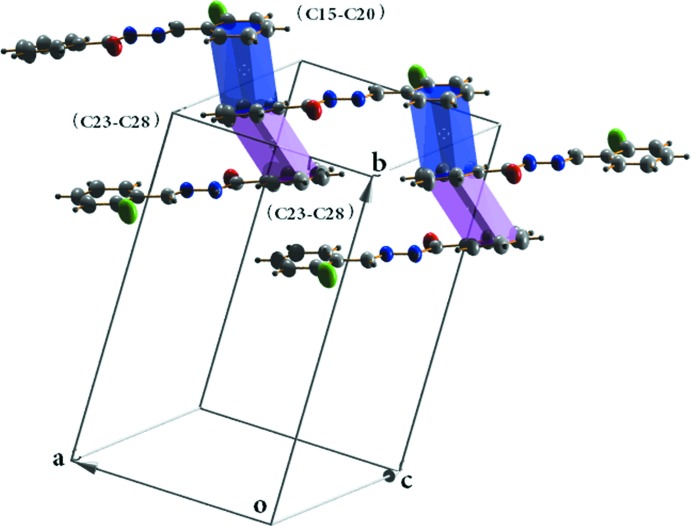
π–π interactions in the title compound.

**Fig. 4.**
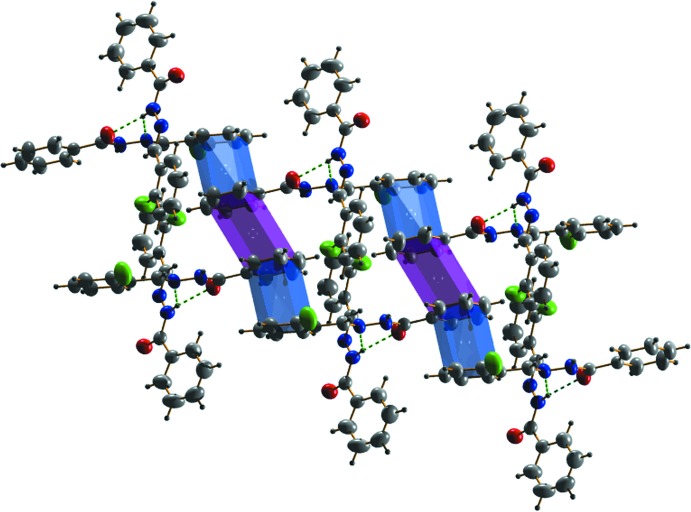
Packing diagram of the title compound along the a axis direction.

## supplementary crystallographic information

### 1. Comment

The coordination chemistry of acyl and aroyl hydrazones have been a subject of competitive research as they are multipurpose class of ligands. Apart from exhibiting physiological and biological activities (Sreeja *et al.*, 2004), they also function as catalysts (Heravi *et al.*, 2007) as well as inhibitors for many enzymes (Tamasi *et al.*, 2005).

The title compound crystallizes in monoclinic space group *P*2_1_/n. The asymmetric unit contains two molecules, both of which adopt the *E* configuration with respect to the C═N bond (Fig. 1). They exist in the amido form with a C8═O1 bond length of 1.231 (2) Å and C22═O2 of 1.223 (2) Å, which are very close to the reported C═O bond length in closely related structure (Nair *et al.*, 2012). Both molecules adopt the *Z* conformation with respect to the amido C—N bonds, with torsion angles of -7.2 (3)° and 5.5 (3)°.

There are eight intermolecular hydrogen bonding interactions, out of which three are classical and the rest of interactions are non-classical with D···A distances of 2.875 (2), 3.339 (2), 2.882 (2), 3.213 (2), 3.402 (3), 3.450 (3), 3.250 (2) and 3.312 (2) Å (Table 1). The hydrogen at the N2 atom forms bifurcated H bonds with the O2 and N3 atoms of the adjacent molecules (Fig. 2). Two π–π interactions between the rings C15—C20 & C23—C28 (shown in blue) and between the two C23—C28 rings (shown in pink) of the adjacent molecules with the intercentroid distances of 3.8840 (14) Å and 3.9145 (14) Å, respectively, also link the molecules (Fig. 3). Fig. 4 shows the packing of the molecules by means of hydrogen bonding and π–π interactions.

### 2. Experimental

The title compound was prepared by adapting a reported procedure (Mangalam & Kurup, 2011). Benzoic acid hydrazide (1 mmol, 0.136 g) was dissolved in methanol and refluxed with methanolic solution of 2-fluorobenzaldehyde (1 mmol 0.124 g), in presence of a few drops of glacial acetic acid for 6 h. On cooling the reactant media, crystals of hydrazone were separated out. The crystals were filtered and washed with minimum quantity of methanol and dried over P_4_O_10_*in vacuo*. Good quality crystals suitable for X-ray analysis were obtained from methanolic solution by slow evaporation.

### 3. Refinement

All H atoms on C were placed in calculated positions, guided by difference maps, with C—H bond distances of 0.93 Å. H atoms were assigned *U*_iso_(H) values of 1.2Ueq(carrier). H atoms of N2—H2' and N4—H4' bonds were located from difference maps and the bond distances are restrained to 0.88±0.01 Å.

### Crystal data

**Table:**

C_14_H_11_FN_2_O	*F*(000) = 1008
*M**_r_* = 242.25	*D*_x_ = 1.310 Mg m^−^^3^
Monoclinic, *P*2_1_/*n*	Mo *K*α radiation, λ = 0.71073 Å
Hall symbol: -P 2yn	Cell parameters from 4473 reflections
*a* = 9.7010 (6) Å	θ = 4.7–48.2°
*b* = 17.4114 (13) Å	µ = 0.10 mm^−^^1^
*c* = 15.002 (1) Å	*T* = 296 K
β = 104.126 (4)°	Block, colourless
*V* = 2457.3 (3) Å^3^	0.35 × 0.30 × 0.25 mm
*Z* = 8	

### Data collection

**Table:**

Bruker Kappa APEXII CCD area-detector diffractometer	6111 independent reflections
Radiation source: fine-focus sealed tube	3320 reflections with *I* > 2σ(*I*)
Graphite monochromator	*R*_int_ = 0.026
Detector resolution: 8.33 pixels mm^-1^	θ_max_ = 28.3°, θ_min_ = 2.5°
ω and φ scan	*h* = −12→11
Absorption correction: multi-scan (*SADABS*; Bruker, 2007)	*k* = −23→17
*T*_min_ = 0.968, *T*_max_ = 0.977	*l* = −19→19
18766 measured reflections	

### Refinement

**Table:**

Refinement on *F*^2^	Primary atom site location: structure-invariant direct methods
Least-squares matrix: full	Secondary atom site location: difference Fourier map
*R*[*F*^2^ > 2σ(*F*^2^)] = 0.050	Hydrogen site location: inferred from neighbouring sites
*wR*(*F*^2^) = 0.165	H atoms treated by a mixture of independent and constrained refinement
*S* = 1.00	*w* = 1/[σ^2^(*F*_o_^2^) + (0.0763*P*)^2^ + 0.4072*P*] where *P* = (*F*_o_^2^ + 2*F*_c_^2^)/3
5972 reflections	(Δ/σ)_max_ = 0.001
333 parameters	Δρ_max_ = 0.21 e Å^−^^3^
2 restraints	Δρ_min_ = −0.17 e Å^−^^3^

### Special details

**Table:**

Geometry. All s.u.'s (except the s.u. in the dihedral angle between two l.s. planes) are estimated using the full covariance matrix. The cell s.u.'s are taken into account individually in the estimation of s.u.'s in distances, angles and torsion angles; correlations between s.u.'s in cell parameters are only used when they are defined by crystal symmetry. An approximate (isotropic) treatment of cell s.u.'s is used for estimating s.u.'s involving l.s. planes.
Refinement. Refinement of *F*^2^ against ALL reflections. The weighted *R*-factor *wR* and goodness of fit *S* are based on *F*^2^, conventional *R*-factors *R* are based on *F*, with *F* set to zero for negative *F*^2^. The threshold expression of *F*^2^ > σ(*F*^2^) is used only for calculating *R*-factors(gt) *etc*. and is not relevant to the choice of reflections for refinement. *R*-factors based on *F*^2^ are statistically about twice as large as those based on *F*, and *R*- factors based on ALL data will be even larger.

### Fractional atomic coordinates and isotropic or equivalent isotropic displacement parameters (Å^2^)

**Table:**

	*x*	*y*	*z*	*U*_iso_*/*U*_eq_	
F1	0.46744 (16)	0.52151 (9)	0.31008 (10)	0.0961 (5)	
F2	0.04425 (16)	0.24110 (10)	0.23115 (9)	0.0973 (5)	
O1	0.42500 (14)	0.14776 (8)	0.21005 (9)	0.0613 (4)	
O2	0.28675 (14)	0.15829 (9)	−0.13577 (9)	0.0630 (4)	
N1	0.44609 (15)	0.29885 (10)	0.23441 (10)	0.0513 (4)	
N2	0.54248 (16)	0.24863 (9)	0.28584 (10)	0.0505 (4)	
N3	0.16870 (15)	0.19149 (9)	0.00186 (10)	0.0490 (4)	
N4	0.30586 (16)	0.16402 (10)	0.01524 (11)	0.0512 (4)	
C1	0.3744 (2)	0.50230 (12)	0.23098 (15)	0.0591 (5)	
C2	0.2916 (3)	0.55868 (13)	0.18222 (19)	0.0736 (6)	
H2	0.3001	0.6092	0.2028	0.088*	
C3	0.1957 (3)	0.53942 (15)	0.1024 (2)	0.0862 (8)	
H3	0.1382	0.5770	0.0680	0.103*	
C4	0.1845 (3)	0.46473 (16)	0.07311 (19)	0.0900 (8)	
H4	0.1194	0.4517	0.0187	0.108*	
C5	0.2685 (2)	0.40901 (13)	0.12344 (15)	0.0706 (6)	
H5	0.2595	0.3585	0.1026	0.085*	
C6	0.36690 (19)	0.42641 (11)	0.20489 (13)	0.0506 (5)	
C7	0.46029 (19)	0.36878 (12)	0.25810 (13)	0.0539 (5)	
H7	0.5304	0.3832	0.3095	0.065*	
C8	0.52872 (18)	0.17362 (11)	0.26640 (11)	0.0464 (4)	
C9	0.64558 (19)	0.12175 (11)	0.31359 (12)	0.0490 (4)	
C10	0.6233 (3)	0.04460 (14)	0.30166 (17)	0.0771 (7)	
H10	0.5351	0.0268	0.2689	0.093*	
C11	0.7297 (3)	−0.00725 (16)	0.33747 (19)	0.0953 (9)	
H11	0.7136	−0.0596	0.3280	0.114*	
C12	0.8586 (3)	0.01828 (16)	0.38681 (18)	0.0840 (8)	
H12	0.9303	−0.0166	0.4118	0.101*	
C13	0.8819 (2)	0.09439 (17)	0.39922 (19)	0.0865 (8)	
H13	0.9699	0.1119	0.4327	0.104*	
C14	0.7759 (2)	0.14642 (14)	0.36265 (16)	0.0711 (6)	
H14	0.7932	0.1988	0.3714	0.085*	
C15	−0.0531 (2)	0.24951 (13)	0.15067 (14)	0.0599 (5)	
C16	−0.1862 (3)	0.27441 (13)	0.15287 (17)	0.0698 (6)	
H16	−0.2083	0.2854	0.2084	0.084*	
C17	−0.2853 (2)	0.28265 (13)	0.07180 (19)	0.0710 (6)	
H17	−0.3766	0.2990	0.0718	0.085*	
C18	−0.2513 (2)	0.26703 (13)	−0.00981 (16)	0.0674 (6)	
H18	−0.3194	0.2730	−0.0650	0.081*	
C19	−0.1169 (2)	0.24265 (12)	−0.01023 (14)	0.0565 (5)	
H19	−0.0948	0.2323	−0.0660	0.068*	
C20	−0.01365 (19)	0.23326 (10)	0.07091 (13)	0.0488 (4)	
C21	0.1288 (2)	0.20552 (11)	0.07427 (13)	0.0514 (5)	
H21	0.1920	0.1979	0.1311	0.062*	
C22	0.35624 (18)	0.14576 (10)	−0.05743 (12)	0.0461 (4)	
C23	0.49959 (18)	0.11042 (10)	−0.03891 (12)	0.0476 (4)	
C24	0.5548 (2)	0.09675 (15)	−0.11316 (16)	0.0715 (6)	
H24	0.5039	0.1104	−0.1719	0.086*	
C25	0.6874 (3)	0.06241 (17)	−0.1000 (2)	0.0918 (8)	
H25	0.7244	0.0524	−0.1505	0.110*	
C26	0.7637 (3)	0.04326 (15)	−0.0146 (2)	0.0873 (8)	
H26	0.8531	0.0210	−0.0067	0.105*	
C27	0.7102 (2)	0.05644 (14)	0.05883 (18)	0.0747 (7)	
H27	0.7626	0.0433	0.1174	0.090*	
C28	0.5777 (2)	0.08945 (12)	0.04711 (14)	0.0604 (5)	
H28	0.5406	0.0976	0.0979	0.073*	
H4'	0.3472 (19)	0.1550 (11)	0.0722 (7)	0.060 (6)*	
H2'	0.6116 (18)	0.2682 (12)	0.3300 (12)	0.077 (7)*	

### Atomic displacement parameters (Å^2^)

**Table:**

	*U*^11^	*U*^22^	*U*^33^	*U*^12^	*U*^13^	*U*^23^
F1	0.0962 (10)	0.0799 (10)	0.0961 (10)	−0.0050 (8)	−0.0077 (9)	−0.0251 (8)
F2	0.0970 (10)	0.1417 (14)	0.0495 (7)	0.0320 (9)	0.0106 (7)	−0.0092 (8)
O1	0.0577 (8)	0.0644 (9)	0.0510 (7)	−0.0008 (6)	−0.0076 (6)	−0.0029 (6)
O2	0.0597 (8)	0.0844 (11)	0.0418 (7)	0.0165 (7)	0.0064 (6)	0.0050 (7)
N1	0.0475 (9)	0.0575 (11)	0.0449 (8)	0.0057 (7)	0.0036 (7)	0.0017 (7)
N2	0.0483 (9)	0.0535 (10)	0.0430 (8)	0.0049 (7)	−0.0015 (7)	−0.0018 (7)
N3	0.0430 (8)	0.0565 (10)	0.0446 (8)	0.0079 (7)	0.0054 (7)	−0.0006 (7)
N4	0.0448 (8)	0.0657 (11)	0.0392 (8)	0.0139 (7)	0.0027 (7)	0.0006 (8)
C1	0.0513 (11)	0.0597 (14)	0.0664 (13)	−0.0037 (9)	0.0146 (10)	−0.0060 (11)
C2	0.0728 (15)	0.0510 (13)	0.1003 (18)	0.0042 (11)	0.0276 (14)	0.0002 (12)
C3	0.0769 (16)	0.0693 (17)	0.102 (2)	0.0155 (13)	0.0025 (15)	0.0153 (15)
C4	0.0893 (18)	0.0762 (18)	0.0841 (17)	0.0092 (14)	−0.0184 (14)	0.0068 (14)
C5	0.0721 (14)	0.0571 (14)	0.0712 (14)	0.0045 (11)	−0.0045 (12)	0.0013 (11)
C6	0.0443 (10)	0.0538 (12)	0.0536 (11)	0.0000 (8)	0.0120 (8)	0.0014 (9)
C7	0.0508 (11)	0.0570 (13)	0.0499 (11)	0.0003 (9)	0.0047 (8)	−0.0034 (9)
C8	0.0449 (10)	0.0579 (12)	0.0339 (9)	0.0026 (8)	0.0049 (7)	−0.0002 (8)
C9	0.0527 (11)	0.0529 (12)	0.0392 (9)	0.0048 (8)	0.0066 (8)	0.0013 (8)
C10	0.0804 (16)	0.0602 (15)	0.0766 (15)	0.0064 (11)	−0.0081 (13)	−0.0036 (12)
C11	0.115 (2)	0.0582 (16)	0.097 (2)	0.0206 (14)	−0.0042 (17)	0.0005 (14)
C12	0.0837 (18)	0.0819 (19)	0.0815 (17)	0.0333 (14)	0.0107 (14)	0.0127 (14)
C13	0.0576 (13)	0.091 (2)	0.0985 (19)	0.0129 (13)	−0.0052 (13)	0.0154 (16)
C14	0.0556 (12)	0.0655 (15)	0.0806 (15)	0.0029 (10)	−0.0058 (11)	0.0068 (12)
C15	0.0647 (13)	0.0636 (13)	0.0505 (11)	0.0088 (10)	0.0124 (10)	−0.0022 (10)
C16	0.0799 (16)	0.0693 (15)	0.0690 (14)	0.0092 (12)	0.0351 (13)	−0.0027 (11)
C17	0.0558 (13)	0.0658 (15)	0.0957 (18)	0.0097 (10)	0.0266 (13)	0.0038 (13)
C18	0.0555 (12)	0.0709 (15)	0.0712 (14)	0.0073 (10)	0.0064 (11)	0.0061 (12)
C19	0.0539 (11)	0.0628 (13)	0.0524 (11)	0.0068 (9)	0.0123 (9)	0.0023 (10)
C20	0.0527 (11)	0.0445 (11)	0.0491 (10)	0.0036 (8)	0.0121 (9)	−0.0005 (8)
C21	0.0500 (10)	0.0604 (12)	0.0407 (10)	0.0085 (9)	0.0052 (8)	0.0004 (9)
C22	0.0465 (10)	0.0460 (11)	0.0436 (10)	0.0009 (8)	0.0070 (8)	0.0003 (8)
C23	0.0442 (10)	0.0446 (11)	0.0535 (11)	−0.0015 (8)	0.0108 (8)	−0.0041 (8)
C24	0.0652 (14)	0.0886 (18)	0.0640 (13)	0.0070 (12)	0.0222 (11)	−0.0062 (12)
C25	0.0751 (17)	0.112 (2)	0.101 (2)	0.0148 (15)	0.0460 (16)	−0.0117 (17)
C26	0.0539 (14)	0.0893 (19)	0.121 (2)	0.0176 (12)	0.0263 (15)	0.0003 (17)
C27	0.0528 (12)	0.0787 (16)	0.0863 (17)	0.0179 (11)	0.0046 (12)	0.0043 (13)
C28	0.0532 (11)	0.0659 (14)	0.0599 (12)	0.0128 (10)	0.0091 (10)	0.0017 (10)

### Geometric parameters (Å, º)

**Table:**

F1—C1	1.346 (2)	C11—H11	0.9300
F2—C15	1.347 (2)	C12—C13	1.350 (4)
O1—C8	1.231 (2)	C12—H12	0.9300
O2—C22	1.223 (2)	C13—C14	1.380 (3)
N1—C7	1.266 (2)	C13—H13	0.9300
N1—N2	1.372 (2)	C14—H14	0.9300
N2—C8	1.338 (2)	C15—C16	1.370 (3)
N2—H2'	0.887 (9)	C15—C20	1.372 (3)
N3—C21	1.263 (2)	C16—C17	1.362 (3)
N3—N4	1.382 (2)	C16—H16	0.9300
N4—C22	1.337 (2)	C17—C18	1.371 (3)
N4—H4'	0.865 (9)	C17—H17	0.9300
C1—C2	1.363 (3)	C18—C19	1.373 (3)
C1—C6	1.375 (3)	C18—H18	0.9300
C2—C3	1.367 (4)	C19—C20	1.384 (3)
C2—H2	0.9300	C19—H19	0.9300
C3—C4	1.368 (4)	C20—C21	1.453 (3)
C3—H3	0.9300	C21—H21	0.9300
C4—C5	1.370 (3)	C22—C23	1.484 (2)
C4—H4	0.9300	C23—C24	1.370 (3)
C5—C6	1.388 (3)	C23—C28	1.377 (3)
C5—H5	0.9300	C24—C25	1.388 (3)
C6—C7	1.453 (3)	C24—H24	0.9300
C7—H7	0.9300	C25—C26	1.356 (4)
C8—C9	1.487 (2)	C25—H25	0.9300
C9—C10	1.365 (3)	C26—C27	1.347 (4)
C9—C14	1.368 (3)	C26—H26	0.9300
C10—C11	1.378 (3)	C27—C28	1.380 (3)
C10—H10	0.9300	C27—H27	0.9300
C11—C12	1.363 (4)	C28—H28	0.9300
			
C7—N1—N2	116.26 (15)	C14—C13—H13	119.8
C8—N2—N1	118.79 (15)	C9—C14—C13	120.6 (2)
C8—N2—H2'	123.7 (15)	C9—C14—H14	119.7
N1—N2—H2'	117.5 (15)	C13—C14—H14	119.7
C21—N3—N4	115.38 (15)	F2—C15—C16	118.13 (19)
C22—N4—N3	119.63 (14)	F2—C15—C20	118.40 (18)
C22—N4—H4'	126.0 (13)	C16—C15—C20	123.5 (2)
N3—N4—H4'	113.9 (13)	C17—C16—C15	118.4 (2)
F1—C1—C2	118.3 (2)	C17—C16—H16	120.8
F1—C1—C6	117.84 (19)	C15—C16—H16	120.8
C2—C1—C6	123.8 (2)	C16—C17—C18	120.3 (2)
C1—C2—C3	118.7 (2)	C16—C17—H17	119.8
C1—C2—H2	120.7	C18—C17—H17	119.8
C3—C2—H2	120.7	C17—C18—C19	120.1 (2)
C2—C3—C4	119.9 (2)	C17—C18—H18	119.9
C2—C3—H3	120.1	C19—C18—H18	119.9
C4—C3—H3	120.1	C18—C19—C20	121.1 (2)
C3—C4—C5	120.4 (2)	C18—C19—H19	119.4
C3—C4—H4	119.8	C20—C19—H19	119.4
C5—C4—H4	119.8	C15—C20—C19	116.53 (18)
C4—C5—C6	121.3 (2)	C15—C20—C21	120.25 (17)
C4—C5—H5	119.4	C19—C20—C21	123.20 (18)
C6—C5—H5	119.4	N3—C21—C20	121.54 (16)
C1—C6—C5	115.95 (18)	N3—C21—H21	119.2
C1—C6—C7	121.65 (18)	C20—C21—H21	119.2
C5—C6—C7	122.37 (19)	O2—C22—N4	121.02 (16)
N1—C7—C6	119.94 (17)	O2—C22—C23	121.70 (17)
N1—C7—H7	120.0	N4—C22—C23	117.27 (15)
C6—C7—H7	120.0	C24—C23—C28	118.78 (18)
O1—C8—N2	121.89 (16)	C24—C23—C22	117.03 (17)
O1—C8—C9	120.36 (18)	C28—C23—C22	124.17 (18)
N2—C8—C9	117.73 (15)	C23—C24—C25	119.5 (2)
C10—C9—C14	118.38 (19)	C23—C24—H24	120.3
C10—C9—C8	117.23 (18)	C25—C24—H24	120.3
C14—C9—C8	124.25 (19)	C26—C25—C24	120.9 (3)
C9—C10—C11	120.9 (2)	C26—C25—H25	119.6
C9—C10—H10	119.5	C24—C25—H25	119.6
C11—C10—H10	119.5	C27—C26—C25	120.0 (2)
C12—C11—C10	119.9 (3)	C27—C26—H26	120.0
C12—C11—H11	120.0	C25—C26—H26	120.0
C10—C11—H11	120.0	C26—C27—C28	120.0 (2)
C13—C12—C11	119.7 (2)	C26—C27—H27	120.0
C13—C12—H12	120.2	C28—C27—H27	120.0
C11—C12—H12	120.2	C23—C28—C27	120.8 (2)
C12—C13—C14	120.5 (2)	C23—C28—H28	119.6
C12—C13—H13	119.8	C27—C28—H28	119.6
			
C7—N1—N2—C8	177.11 (17)	C12—C13—C14—C9	0.3 (4)
C21—N3—N4—C22	178.37 (18)	F2—C15—C16—C17	−179.7 (2)
F1—C1—C2—C3	−179.3 (2)	C20—C15—C16—C17	0.8 (4)
C6—C1—C2—C3	−0.3 (4)	C15—C16—C17—C18	−0.7 (4)
C1—C2—C3—C4	0.0 (4)	C16—C17—C18—C19	0.3 (4)
C2—C3—C4—C5	0.2 (5)	C17—C18—C19—C20	0.1 (3)
C3—C4—C5—C6	−0.1 (4)	F2—C15—C20—C19	179.99 (19)
F1—C1—C6—C5	179.36 (19)	C16—C15—C20—C19	−0.5 (3)
C2—C1—C6—C5	0.4 (3)	F2—C15—C20—C21	1.8 (3)
F1—C1—C6—C7	−2.7 (3)	C16—C15—C20—C21	−178.7 (2)
C2—C1—C6—C7	178.3 (2)	C18—C19—C20—C15	0.0 (3)
C4—C5—C6—C1	−0.2 (3)	C18—C19—C20—C21	178.2 (2)
C4—C5—C6—C7	−178.1 (2)	N4—N3—C21—C20	−178.78 (17)
N2—N1—C7—C6	177.19 (16)	C15—C20—C21—N3	−178.67 (19)
C1—C6—C7—N1	176.73 (19)	C19—C20—C21—N3	3.3 (3)
C5—C6—C7—N1	−5.5 (3)	N3—N4—C22—O2	5.5 (3)
N1—N2—C8—O1	−7.2 (3)	N3—N4—C22—C23	−174.97 (16)
N1—N2—C8—C9	171.27 (15)	O2—C22—C23—C24	4.0 (3)
O1—C8—C9—C10	−9.0 (3)	N4—C22—C23—C24	−175.59 (18)
N2—C8—C9—C10	172.51 (19)	O2—C22—C23—C28	−174.60 (19)
O1—C8—C9—C14	166.5 (2)	N4—C22—C23—C28	5.8 (3)
N2—C8—C9—C14	−12.0 (3)	C28—C23—C24—C25	−0.1 (3)
C14—C9—C10—C11	−0.5 (4)	C22—C23—C24—C25	−178.8 (2)
C8—C9—C10—C11	175.3 (2)	C23—C24—C25—C26	−0.9 (4)
C9—C10—C11—C12	1.0 (4)	C24—C25—C26—C27	1.0 (4)
C10—C11—C12—C13	−0.9 (4)	C25—C26—C27—C28	0.0 (4)
C11—C12—C13—C14	0.3 (4)	C24—C23—C28—C27	1.1 (3)
C10—C9—C14—C13	−0.1 (4)	C22—C23—C28—C27	179.6 (2)
C8—C9—C14—C13	−175.6 (2)	C26—C27—C28—C23	−1.0 (4)

### Hydrogen-bond geometry (Å, º)

**Table:**

*D*—H···*A*	*D*—H	H···*A*	*D*···*A*	*D*—H···*A*
N2—H2′···O2^i^	0.89 (1)	2.09 (2)	2.875 (2)	147 (2)
N2—H2′···N3^i^	0.89 (1)	2.60 (2)	3.339 (2)	142 (2)
N4—H4′···O1	0.87 (1)	2.03 (1)	2.882 (2)	171 (2)
C7—H7···O2^i^	0.93	2.53	3.213 (2)	131
C14—H14···O2^i^	0.93	2.49	3.402 (3)	166
C16—H16···O2^ii^	0.93	2.55	3.450 (3)	164
C21—H21···O1	0.93	2.44	3.250 (2)	145
C28—H28···O1	0.93	2.40	3.312 (2)	166

Symmetry codes: (i) *x*+1/2, −*y*+1/2, *z*+1/2; (ii) *x*−1/2, −*y*+1/2, *z*+1/2.
